# Allosteric Regulation of the Human and Mouse Deoxyribonucleotide Triphosphohydrolase Sterile α-Motif/Histidine-Aspartate Domain-containing Protein 1 (SAMHD1)*

**DOI:** 10.1074/jbc.M114.571091

**Published:** 2014-05-14

**Authors:** Cristina Miazzi, Paola Ferraro, Giovanna Pontarin, Chiara Rampazzo, Peter Reichard, Vera Bianchi

**Affiliations:** From the Department of Biology, University of Padova, 35131 Padova, Italy

**Keywords:** Allosteric Regulation, DNA Enzyme, DNA Replication, Enzyme Kinetics, Enzyme Mechanism

## Abstract

The deoxyribonucleotide triphosphohydrolase SAMHD1 restricts lentiviral infection by depleting the dNTPs required for viral DNA synthesis. In cultured human fibroblasts SAMHD1 is expressed maximally during quiescence preventing accumulation of dNTPs outside S phase. siRNA silencing of SAMHD1 increases dNTP pools, stops cycling human cells in G_1_, and blocks DNA replication. Surprisingly, knock-out of the mouse gene does not affect the well being of the animals. dNTPs are both substrates and allosteric effectors for SAMHD1. In the crystal structure each subunit of the homotetrameric protein contains one substrate-binding site and two nonidentical effector-binding sites, site 1 binding dGTP, site 2 dGTP or dATP. Here we compare allosteric properties of pure recombinant human and mouse SAMHD1. Both enzymes are activated 3–4-fold by allosteric effectors. We propose that in quiescent cells where SAMHD1 is maximally expressed GTP binds to site 1 with very high affinity, stabilizing site 2 of the tetrameric structure. Any canonical dNTP can bind to site 2 and activate SAMHD1, but in cells only dATP or dTTP are present at sufficient concentrations. The apparent *K_m_* for dATP at site 2 is ∼10 μm for mouse and 1 μm for human SAMHD1, for dTTP the corresponding values are 50 and 2 μm. Tetrameric SAMHD1 is activated for the hydrolysis of any dNTP only after binding of a dNTP to site 2. The lower *K_m_* constants for human SAMHD1 induce activation at lower cellular concentrations of dNTPs thereby limiting the size of dNTP pools more efficiently in quiescent human cells.

## Teaser

**Background:** Genetic loss of SAMHD1, a catabolic enzyme preventing dNTP accumulation outside S phase, leads to disease in humans but not in mice.

**Results:** Allosteric regulation by dNTPs works similarly in pure recombinant human and mouse SAMHD1.

**Conclusion:** Human SAMHD1 is more efficiently regulated at low dNTP concentrations.

**Significance:** Inside cells dATP and dTTP are the main regulators of both human and mouse SAMHD1.

## Introduction

SAMHD1^3^ is a mammalian triphosphohydrolase that transforms dNTPs to the corresponding deoxyribonucleosides + triphosphate. Various eukaryotes as well as eubacteria and archea contain orthologues of SAMHD1. Interest in the protein exploded in recent years, when it was discovered that the enzyme via its phosphohydrolase activity (1, 2) serves as a restriction factor during HIV-1 infection (3, 4). Additionally, mutations in SAMHD1 were found to cause Aicardi-Goutières syndrome, a rare genetic autoimmune disease (5).

The name SAMHD1 was coined from its protein structure that contains two previously known modules: an N-terminal SAM domain preceded by a nuclear localization signal and followed by a catalytic HD domain plus a disordered ≈40 amino acid-long C terminus. The complete protein as well as the isolated HD domain show triphosphohydrolase activity (1, 2, 6, 7) and provide HIV-1 restriction by depleting the host cells of the dNTPs required for the synthesis of the viral DNA. The SAM domain is a putative protein-protein or protein-nucleic acid interaction module (8) with an unclear function in SAMHD1.

Closely related triphosphohydrolases had been described earlier in microorganisms and in pure form were characterized in considerable detail (9–12). It was suggested that these enzymes participate in the regulation of dNTP pool sizes and that their action prohibits an undue accumulation of dNTPs that would interfere with the ordinary replication of DNA (11). Detailed studies of the kinetic properties and structures of the triphosphohydrolases from *Thermus thermophilus*, *Pseudomonas*, and *Enterococcus faecalis* demonstrated that dNTPs not only are substrates for the enzymes but also work as allosteric effectors. Their crystal structures revealed a hexameric homo-oligomer as the biologically active unit of the *T. thermophilus* triphosphohydrolase (12) and a tetrameric homo-oligomer for the *E. faecalis* enzyme (10), with distinct binding sites for substrates and effectors.

After a first report that a crystallized shortened form (amino acids 120–626) of SAMHD1 is enzymatically active and has a dimeric structure (1), two recent closely spaced publications described structures of slightly longer catalytic cores of the human protein, starting at residue 113 or 109, in complex with dGTP or dGTP/dATP, respectively (13, 14). In both cases dGTP induced the formation of a catalytically active tetramer with four substrate binding sites and eight allosteric sites. Two classes of allosteric sites could be distinguished: site 1, binding exclusively dGTP; and site 2, binding dGTP or dATP. The structure shows that each effector-binding site results from the interaction of three subunits in the tetrameric protein. So far only a few laboratories have studied the biochemical properties of purified full-length or truncated SAMHD1 (1, 2, 7, 15). The enzyme removed the triphosphate moiety of a dNTP and was marginally active with rNTPs. Both the human and mouse enzymes were highly active with dGTP but scarcely active with other dNTPs unless dGTP was present as an activator. At close to physiological concentrations, GTP was more effective than dGTP in stimulating dATP hydrolysis and was suggested to be the physiological primary activator of SAMHD1 in cells (7). The enzyme presented nuclease activity, with still unknown physiological implications (15).

Our own work has for a long time been concerned with the enzymes that in mammalian cells regulate the synthesis and degradation of dNTPs during the cell cycle in relation to DNA replication. We found that the concentration of the dNTPs both in the cytosol and in mitochondria depends not only on their synthesis by ribonucleotide reductase, deoxyribonucleoside, and deoxynucleotide kinases and their consumption by DNA replication and repair, but also on their continuous degradation by nucleotidases and other catabolic enzymes (16). With the appearance of SAMHD1 we suspected that this enzyme participates in the “tug of war” between anabolism and catabolism. In cultured human fibroblasts we indeed found that SAMHD1 was variably expressed during the cell cycle, maximally during quiescence and minimally during S phase (17). In cycling cells siRNA silencing of SAMHD1 was accompanied by expansion and imbalance of dNTP pools with the cells accumulating in the G_1_ phase. After removal of the siRNA the pools progressively normalized and growth restarted, suggesting that in proliferating cells SAMHD1 limits dNTP pool size during G_1_ before entry into S phase. A similar arrest in G_1_ had been reported earlier in yeast cells with constitutively high concentrations of dNTPs (18).

The SAMHD1 gene is present in many organisms and expressed in most tissues (19), suggesting a general function of the protein for the maintenance of small dNTP pools outside S phase. This concept was challenged by two recent reports suggesting that knock-out of the mouse gene had no effect on the well being of the animals and did not reproduce the Aicardi-Goutières phenotype (20, 21). Possibly other mechanisms might substitute for SAMHD1, or the mouse tolerates larger dNTP pools better than humans.

In a first attempt to explain the difference we here compare the kinetic properties of full-length mouse and human SAMHD1 with emphasis on their allosteric regulation. The two enzymes had similar *K_m_* constants for the hydrolysis of each of the four canonical dNTPs, but the *k*_cat_ constants were 2–4 times higher for the mouse enzyme, indicating a somewhat higher turnover. Considering the structure of SAMHD1, the kinetic properties suggest that allosteric site 1 exclusively binds GTP or dGTP with *K_m_* values 3 orders of magnitude lower than the *K_m_* values for substrates. Allosteric site 2 binds all four dNTPs but not GTP. With our experiments we could only measure the apparent effector *K_m_* values for dATP and dTTP, both smaller than the substrate *K_m_* values of the two nucleotides. Interestingly, the *K_m_* values for both allosteric sites of the human enzyme were smaller than those of the mouse enzyme, suggesting that human SAMHD1 degrades small dNTP pools more effectively than mouse SAMHD1.

## EXPERIMENTAL PROCEDURES

### 

#### 

##### Materials

^3^H-Labeled dNTPs were from PerkinElmer Life Sciences with an original isotopic purity of >98% and were stored at −80 °C. Their decomposition on storage was checked continuously by the ion exchange procedure described below for the dephosphorylation assay. They were used until their purity was <95%. Nonlabeled dNTPs were from Sigma. The AG1-X2 ion exchange resin was from Bio-Rad.

##### Expression and Purification of Recombinant SAMHD1

**FIGURE 1.**
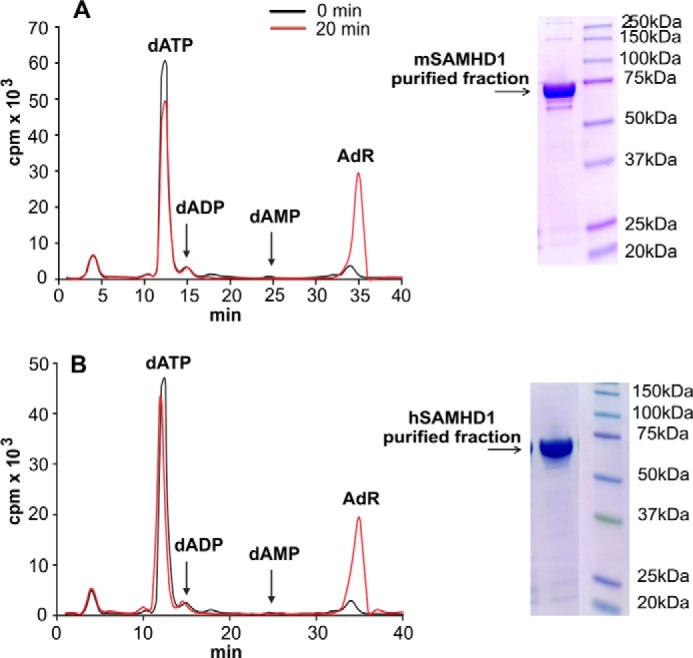
**SAMHD1 is a dNTP triphosphohydrolase.** PAGE analysis and activity assay of purified recombinant mouse and human SAMHD1 are shown. *A*, [^3^H]dATP (100 μm, 30,000 cpm/nmol) and 100 ng of mouse SAMHD1 were incubated for 0 or 20 min under assay conditions. After protein precipitation the reaction mixture was chromatographed isocratically together with nonlabeled carrier deoxyadenosine and dATP as detailed under “Experimental Procedures.” The *graph* shows the radioactivity of the reaction products in 0.5-ml fractions collected at the two time points. *B*, the same experiment is shown but with 300 ng of human SAMHD1. The *insets* show the electrophoretic analyses of 4 μg of each enzyme and the positions of molecular size markers.

We are grateful to Dr. F. Perrino for the gift of the plasmid for mouse SAMHD1 (2), coding for isoform 1 of the enzyme (amino acids 31–658) and to Dr. A. Yakunin for the plasmid coding for full-length human SAMHD1 (15), both containing a N-terminal His tag. SAMHD1 was expressed and overproduced in *Escherichia coli* Rosetta (DE3). The crude bacterial extract was adsorbed to a 1-ml column of nickel-nitrilotriacetic acid resin and purified by stepwise elution with imidazole buffers of increasing imidazole concentrations. Enzyme purity was checked at each step. The material eluting at 100 or 250 mm imidazole was divided in small aliquots, frozen at liquid nitrogen temperature, and used for the experiments. Fig. 1 shows the purity of the preparations by denaturing electrophoresis on Mini-PROTEAN TGX^TM^ Any kDa^TM^ precast gels (Bio-Rad).

##### Assays of the Dephosphorylation of dNTPs

**FIGURE 2.**
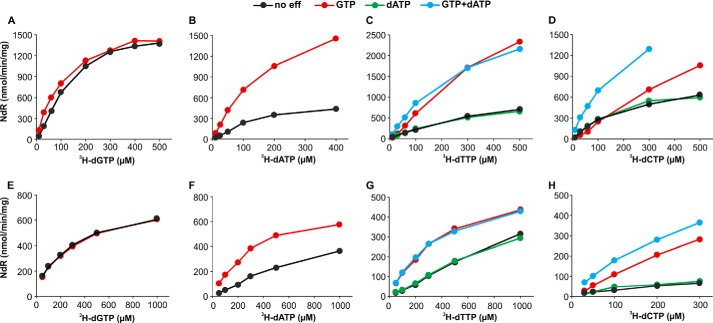
**Influence of substrate concentration on the dephosphorylation of ^3^H-labeled dNTPs by SAMHD1.** *Upper row*, incubations with 50 ng of mouse SAMHD1; *lower row*, incubations with 150 ng of human SAMHD1. Each enzyme was incubated in separate experiments with ^3^H-labeled dGTP, dATP, dTTP, or dCTP (200–6000 cpm/nmol) at the varying concentrations shown on the *abscissa* and with 20 μm effectors (*color-coded* in the *graphs*). Other incubation conditions, the quantitation of the deoxynucleosides formed during the reaction, and the calculation of *K_m_* constants are described under “Experimental Procedures.” *Ordinates* indicate the nmol of deoxynucleoside (*NdR*) produced in 1 min by 1 mg of protein.

A typical experiment consisted of separate parallel tubes containing in a final volume of 0.05 ml, the different concentrations of one [^3^H]dNTP (1,000–20,000 cpm/nmol), various effector combinations, and 20 mm Tris-HCl, pH 7.5, 5 mm MgCl_2_, 2 mm DTT, 50 mm NaCl, 2.5 mm mercaptoethanol, 1 mg/ml BSA, and 12.5% glycerol. After addition of SAMHD1 (50 ng of mouse enzyme or 150 ng of human enzyme) the tubes were incubated at 37 °C for 20 min, the reaction was stopped with 1 ml of 5 mm EDTA, and the solution was passed through a 1-ml column of AG1-X2 ion exchange resin to retain unreacted nucleotides. The produced isotopic deoxyribonucleoside was eluted with 4.5 ml of 50 mm acetic acid. From the total radioactivity in the combined run-through fraction + eluant and the specific radioactivity of the dNTP used for the reaction we calculated the dephosphorylation rate (nmol of deoxyribonucleoside/min/mg of enzyme) shown on the ordinate in most figures. In all cases <15% of the labeled dNTP was dephosphorylated during the incubation. We determined the *K_m_* constant for a substrate from incubations at 20 μm effector (GTP or dATP) concentration (Fig. 2). To determine the *K_m_* constant for an effector (Figs. and 4) we performed incubations at a fixed substrate concentration that minimizes interference with the effector at allosteric site 2 at the same time permitting detection of substrate hydrolysis. For effector *K_m_* values the ordinates show values for *V* − *V_o_*, where *V* is the rate of dephosphorylation in the presence of effector and *V_o_* the rate in its absence. We fitted the kinetic data with the Michaelis-Menten equation using Prism software (version 4.03, GraphPad Software, La Jolla CA). Typically we conducted with each dNTP at least three independent experiments and in Tables 1 and 2 report mean *K_m_* values ± S.E. For constants derived from single experiments we report in the tables the S.E. of the curve fitting provided by the software. We calculated *k*_cat_ constants (turnover number) by dividing the *V*_max_ values obtained from the Michaelis-Menten equation by 60 and by the nanomoles of SAMHD1 subunits used in the individual experiments. Graphs show results from representative experiments.

##### Specificity of SAMHD1 as a Phosphohydrolase

To test the specific hydrolysis of dATP to deoxyadenosine and triphosphate by SAMHD1 (Fig. 1), [^3^H]dATP (100 μm, 30,000 cpm/nmol) and 100 ng of recombinant mouse or 300 ng of human SAMHD1 was incubated for 0 or 20 min under assay conditions and chromatographed isocratically by HPLC together with nonlabeled carrier deoxyadenosine and dATP on a nucleosil C18 column. Flow rate was 1 ml/min for 30 min with 0.2 m NH_4_H_2_PO_4_, pH 3.5, followed by a 20-min gradient to 30% methanol. The reaction products were identified from their UV absorption, and their radioactivity was measured by counting 0.5-ml fractions of the eluate.

## RESULTS

### 

#### 

##### Characterization of Mouse and Human SAMHD1

The two triphosphohydrolases were purified to homogeneity by standard procedures after expression in *E. coli*. On denaturing gels each enzyme gave a single band at the position expected for the monomer (Fig. 1) and during incubation with radioactive dATP exclusively produced labeled deoxyadenosine, demonstrating the high specificity of the enzyme for the hydrolysis of the α-phosphate bond (Fig. 1). This specificity made it possible to construct a simple and highly accurate radioactivity-based assay for the dephosphorylation of each labeled dNTP as described under “Experimental Procedures.”

##### Mouse and Human SAMHD1 Have Similar K_m_ Constants for Substrates but Different k_cat_ Constants

Earlier work demonstrated that the transformation of individual dNTPs to their deoxyribonucleosides by SAMHD1 was strongly stimulated by dGTP or GTP (7). In the experiments of Fig. 2 we measured the influence of substrate concentration of each dNTP on the rate of its dephosphorylation and the stimulation by GTP and other allosteric effectors. The *upper row* in Fig. 2 shows results obtained with the mouse enzyme, the *lower row* results with the human enzyme. In both cases we observed an inherent activity also in the absence of effectors, differing from earlier results by other investigators that had found an almost complete dependence of the reaction on the presence of dGTP (1, 6).

The hydrolysis of purine dNTPs appeared more straightforward than that of pyrimidine dNTPs where attainment of the highest activity required more than one effector. dGTP presented the simplest case as the nucleotide efficiently stimulated its own dephosphorylation. Mouse SAMHD1 at concentrations of dGTP below 100 μm was slightly stimulated by GTP, but not at higher concentrations of substrate (Fig. 2*A*). The human enzyme was not stimulated by GTP at any concentration of dGTP (Fig. 2*E*). The activation of dATP hydrolysis was also simple, with either dGTP or GTP stimulating dephosphorylation by both the mouse and human enzymes (Fig. 2, *B* and *F*). A more detailed comparison of GTP and dGTP as effectors is presented in the next section. Turning to the dephosphorylation of pyrimidine dNTPs, we found that GTP together with dATP gave optimal stimulation. GTP alone strongly stimulated the dephosphorylation of dTTP by both enzymes (Fig. 2, *C* and *G*) and that of dCTP by human SAMHD1 (Fig. 2*H*). With the mouse enzyme, however, hydrolysis of dCTP at concentrations ≤100 μm was slightly but consistently inhibited when GTP was the only effector (Fig. 2*D*). When we tested at tighter intervals more dCTP concentrations we observed that above 120 μm substrate the hydrolysis started to be stimulated by GTP not reaching a plateau even at 1500 μm (data not shown). At all concentrations of dCTP as substrate, dATP (or dTTP, see below) in combination with GTP further stimulated the reaction (Fig. 2*D*).

Both purine and pyrimidine dNTPs showed similar reaction kinetics, with *K_m_* constants for all four substrates varying between 120 and 340 μm, but with *k*_cat_ constants between 2 and 4 nmol · s^−1^/nmol of mouse SAMHD1 and 1 nmol · s^−1^/nmol of human enzyme (Table 1). During the cell cycle the physiological dNTP concentrations rarely exceed 100 μm, and in quiescent cells are often below 1 μm, far below dNTP *K_m_* values as substrates for SAMHD1.

**TABLE 1:** **Kinetic constants for substrate binding by mouse and human SAMHD1** The experiments measured substrate *K_m_* constants and *k*_cat_ values for the dephosphorylation of increasing concentrations of dNTPs at the indicated effector concentrations. GTP was the primary effector in all incubations, dATP was present as secondary effector in the experiments with dTTP or dCTP. *K_m_* and *k*_cat_ are mean values from three or more experiments ± S.E. or single determinations (indicated by *). In the latter case the reported variation is the S.E. of the curve fitting.

Substrate	Effectors		Mouse SAMHD1		Human SAMHD1	
	GTP	dATP	*K_m_*	*k*_cat_*^a^*	*K_m_*	*k*_cat_*^a^*
	μ*m*	μ*m*	μ*m*		μ*m*	
dGTP	20		220 ± 20*	2.9	240 ± 32*	0.9
	500		120 ± 7*	2.2		
dATP	20		220 ± 44*	4.4	340 ± 40*	1.0
	100		230 ± 12*	2.9		
dTTP	20	20	280 ± 32	4.1 ± 0.3	300 ± 49	1 ± 0.2
dCTP	20	20	200 ± 9	2.2 ± 0.5	290 ± 31	0.9 ± 0.1

*^a^ k*_cat_ values were calculated as nmol of product/s/nmol of enzyme.

##### K_m_ Constants for Allosteric Effectors

The data in Fig. 2 are from experiments in which the substrate concentration was varied in the presence of an excess of effector. In the following experiments we varied the effector concentration at constant substrate concentration to evaluate the affinities of the allosteric sites for the effectors from their *K_m_* constants. In these experiments GTP was always present because this nucleotide, by binding to allosteric site 1, induces the tetramerization of SAMHD1 (22) required for the formation of site 2 and the binding of the other effectors (14).

**FIGURE 3.**
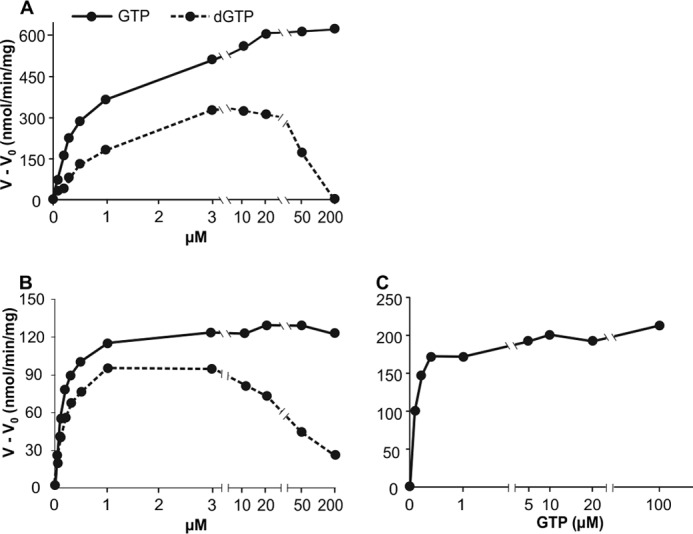
**GTP and dGTP as effectors for the dephosphorylation of dATP and dTTP.** *Upper row*, mouse SAMHD1; *lower row*, human SAMHD1. *A*, and *B*, dephosphorylation of 100 μm [^3^H]dATP by 50 ng of mouse SAMHD1 (*A*) or 150 ng of human SAMHD1 (*B*), at the GTP or dGTP concentrations shown on the abscissa. *C*, dephosphorylation of 100 μm [^3^H]dTTP by 150 ng of human SAMHD1. General conditions are as in Fig. 2. *Ordinates* show *V* − *V_o_* where *V* is the rate of dephosphorylation in the presence of effector and *V_o_* is the rate in its absence.

We begin with the effectors GTP and dGTP in their stimulation of dATP hydrolysis by mouse and human SAMHD1 (Fig. 3, *A* and *B*). With both enzymes low concentrations of either effector stimulated the reaction maximally. The effector *K_m_* constants of the mouse enzyme were 0.5 μm for GTP and 0.8 μm for dGTP, the corresponding values of the human enzyme were 0.15 μm (Table 2). GTP not only had a slightly higher affinity than dGTP for the mouse enzyme but also produced a larger maximal activation (Fig. 3, *A* and *B*). At the low effector concentrations used for the calculations of the effector *K_m_* values, a competition between substrate and effector sites for dGTP binding can be excluded. At higher concentrations competition became apparent for dGTP, but not for GTP, showing that GTP does not bind to the substrate site.

**TABLE 2:** ***K_m_* constants for binding of effectors by mouse and human SAMHD1** The experiments measured binding of increasing concentrations of effectors to site 2 in the presence of a fixed concentration of substrate and of GTP (binding to site 1). Effector *K_m_* values are the mean ± S.E. from three or more identical experiments or from a single experiment (indicated by *) for which the indicated variability is the S.E. of the curve fitting.

Mouse SAMHD1					Human SAMHD1				
Effector	GTP	Substrate	*K_m_*		Effector	GTP	Substrate	*K_m_*	
			Site 1	Site 2				Site 1	Site 2
	μ*m*		μ*m*	μ*m*		μ*m*		μ*m*	μ*m*
GTP		dATP	0.5 ± 0.1		GTP		dATP	0.15 ± 0.01*	
dGTP			0.8 ± 0.1		dGTP			0.14 ± 0.03*	
dATP	20	dTTP		2.5 ± 0.4	GTP		dTTP	0.10 ± 0.01*	
	500			1.5 ± 0.1					
dATP	20	dCTP		11 ± 1.3	dATP	20	dCTP		1.2 ± 0.2
	500			8 ± 1		500			0.9 ± 0.4*
dTTP	20	dCTP		50 ± 1.2	dTTP	20	dCTP		2.1 ± 0.6

GTP was also the major effector for the hydrolysis of dTTP by the human enzyme (see Figs. 2*G* and 3*C*). At 100 μm dTTP, a concentration at which dTTP bound both the catalytic site and allosteric site 2, the effector *K_m_* constant for GTP was 0.15 μm, similar to the *K_m_* of GTP during the hydrolysis of dATP (Table 2).

**FIGURE 4.**
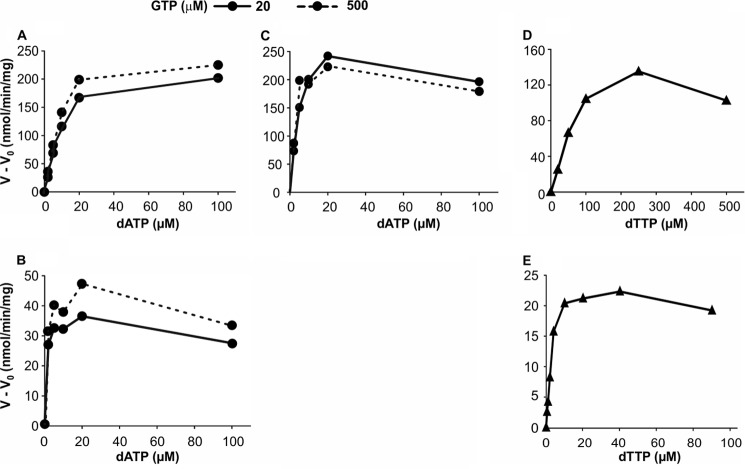
**dATP and dTTP as effectors for the dephosphorylation of dCTP or dTTP in the presence of GTP.** *Upper row*, mouse SAMHD1; *lower row*, human SAMHD1. *A* and *B*, dephosphorylation of 30 μm [^3^H]dCTP at the dATP concentrations shown on the *abscissa* and in the presence of 20 or 500 μm GTP and 50 ng of mouse SAMHD1 (*A*) or 150 ng of human SAMHD1 (*B*), as described in Fig. 2. *C*, hydrolysis of 30 μm [^3^H]dTTP by 50 ng of mouse SAMHD1 with dATP as effector at the above two concentrations of GTP. *D* and *E*, dTTP as effector for the hydrolysis of 30 μm [^3^H]dCTP by 50 ng of mouse SAMHD1 (*D*) or by 150 ng of human enzyme (*E*) in the presence of 20 μm GTP.

dATP worked as an effector for the hydrolysis of dCTP by both the mouse and human enzymes (Fig. 4, *A* and *B*). In the presence of 20 μm GTP, the effector *K_m_* of dATP was 11 μm for mouse SAMHD1 and 1.2 μm for the human enzyme. Increasing the GTP concentration to 500 μm had little effect. The mouse enzyme now had an effector *K_m_* for dATP of 8 μm, the human enzyme 0.9 μm. By testing the two concentrations of GTP we show that GTP does not compete with dATP at allosteric site 2.

dATP also stimulated the hydrolysis of dTTP by mouse SAMHD1 when GTP was present (Fig. 4*C*, see also Fig. 2*C*), with a *K_m_* constant of 2.5 μm at 20 μm GTP and 1.5 at 500 μm GTP (Table 2). We could not determine an effector constant for dATP with dTTP as substrate of the human enzyme because GTP alone provided full stimulation and dATP had no further effect (Fig. 2*G*).

dTTP efficiently activated the hydrolysis of dCTP by the human enzyme (Fig. 4*E*) working as effector in the presence of GTP with a *K_m_* constant of 2.1 μm. The corresponding activation by the mouse enzyme (Fig. 4*D*) occurred with a 20-fold higher *K_m_* of 50 μm (Table 2).

All calculated effector *K_m_* values are summarized in Table 2. All *K_m_* constants at allosteric site 2 are considerably higher for the mouse than the corresponding human values, suggesting that human SAMHD1 is activated at lower dNTP concentrations. Furthermore, all constants are within the range reported for the concentrations of dNTPs in mammalian cells (23) and therefore suitable to regulate SAMHD1 activity.

##### Cooperation and Competition of Effectors at Allosteric Sites

The structural work on SAMHD1 defined two separate allosteric sites from co-crystallizations of the enzyme with dATP and dGTP: one site bound exclusively dGTP, the second site bound either dGTP or dATP (14). Similar experiments were not done with GTP, dTTP, or dCTP. Here we further characterize functional differences between the two sites of mouse and human SAMHD1 from the cooperation and competition between effectors during dephosphorylation of dNTPs.

**FIGURE 5.**
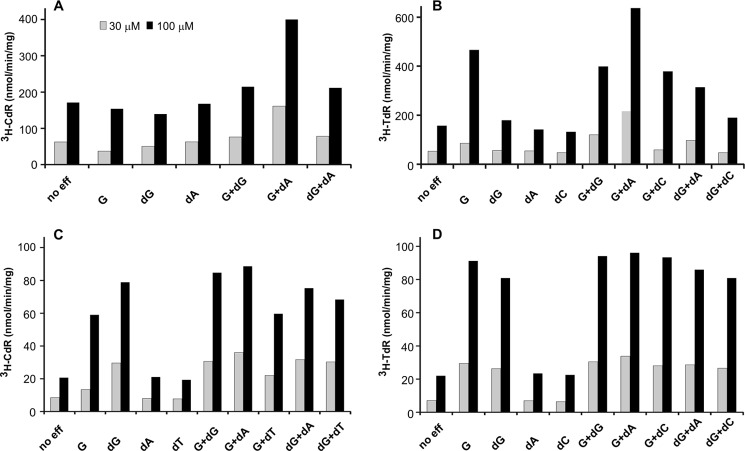
**Cooperation between effectors during the dephosphorylation of dCTP or dTTP by SAMHD1.** *Upper row*, mouse SAMHD1; *lower row*, human SAMHD1. *A*, [^3^H]dCTP as substrate. *B*, [^3^H]dTTP as substrate. We incubated each [^3^H]dNTP (30 μm = *shaded bars*, 100 μm = *black bars*) with 50 ng of mouse SAMHD1 and the indicated effectors (*no eff*, no effector added; *G*, GTP; *dG*, dGTP; *dA*, dATP; *dC*, dCTP; *dT*, dTTP) 10 μm each to determine the formation of labeled deoxycytidine ([*^3^H*]*CdR*) or thymidine (*^3^H-TdR*). General conditions were as described for Fig. 2. The *ordinate* shows the initial rate of each reaction (nmol of deoxyribonucleoside/min/mg of enzyme). *C* and *D*, the corresponding experiments with human SAMHD1, 150 ng/reaction, and all effectors at 3 μm.

We analyzed with the mouse enzyme different combinations of effectors, each at the fixed concentration of 10 μm. Two different concentrations of dCTP (Fig. 5*A*) or dTTP (Fig. 5*B*) (30 and 100 μm) served as substrates. In all instances the combination of GTP and dATP gave the highest activation, agreeing with the results in Fig. 2. All effectors when tested alone did not enhance the dephosphorylation of dCTP by mouse SAMHD1. During dTTP hydrolysis (Fig. 5*B*) dGTP alone produced a small stimulation only at 100 μm dTTP, and dCTP alone was inactive as effector and it did not potentiate the stimulation by GTP. Finally, the combination of GTP with dGTP resulted in a stimulation of dTTP hydrolysis intermediate between those observed with each nucleotide by itself suggesting a competition between the two effectors.

Similar experiments with human SAMHD1 but with all effectors at a concentration of 3 μm are shown in Fig. 5*C* (30 and 100 μm [^3^H]dCTP) and Fig. 5*D* (30 and 100 μm [^3^H]dTTP). Again, the combination of GTP and dATP gave the best activation with both substrates, but now GTP and dGTP alone were similarly active and strongly stimulated the hydrolysis of dCTP (Fig. 5*C*), in contrast to what was observed in Fig. 5*A* with mouse SAMHD1. The difference between the two enzymes may be explained by dCTP not only being a substrate for SAMHD1 but also an effector that stimulates its own hydrolysis as is the case for dTTP (see Fig. 3*C*). If, in analogy to dTTP, dCTP as effector has a much lower *K_m_* constant for human than for mouse SAMHD1, the contrast between Fig. 5, *A* and *C*, is resolved.

**FIGURE 6.**
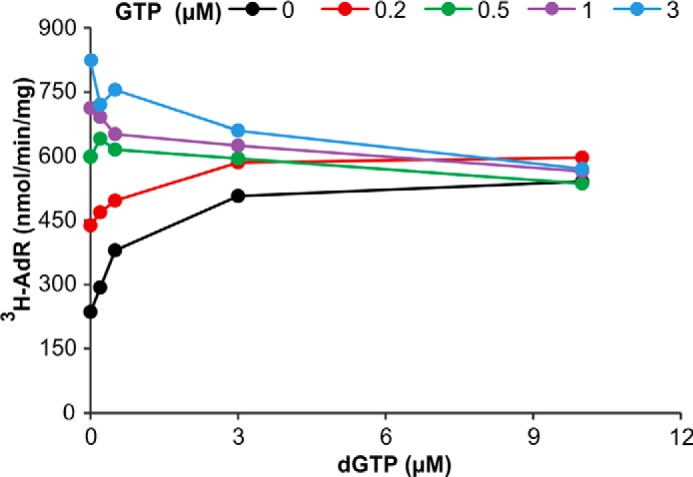
**Competition between dGTP and GTP for allosteric site 1 during dephosphorylation of dATP by mouse SAMHD1.** The experiment shows the changes in SAMHD1 activity by combining increasing concentrations of dGTP with a series of fixed concentrations of GTP as effectors for the dephosphorylation of 30 μm [^3^H]dATP by 50 ng of mouse SAMHD1. The concentration of GTP used in each curve is *color-coded*, the *abscissa* indicates the concentrations of dGTP. The data demonstrate the superiority of GTP as effector and the competition between dGTP and GTP at allosteric site 1. General conditions are as described for Fig. 2.

In the next experiment we investigated in more detail the competition between the two guanine nucleotides at allosteric site 1 of the mouse enzyme by using different combinations of GTP and dGTP to activate the hydrolysis of dATP (Fig. 6). To avoid competition between dATP and dGTP at the substrate site and at allosteric site 2 we employed effector (dGTP) concentrations ≤10 μm, and a high concentration of dATP, 100 μm, that saturated site 2. Thus, we limited the competition between GTP and dGTP to site 1. Raising the concentration of dGTP from 0 to 10 μm in the absence of GTP or with <0.5 μm GTP stimulated the hydrolysis of dATP. However, at concentrations of GTP ≥0.5 μm the addition of dGTP inhibited the reaction, and the degree of inhibition depended on the relative proportions of the two effectors (Fig. 6). As shown in Fig. 3*A*, GTP is a better activator for the hydrolysis of dATP compared with dGTP. The data in Fig. 6 indicate a competition between the two guanine nucleotides for allosteric site 1.

**FIGURE 7.**
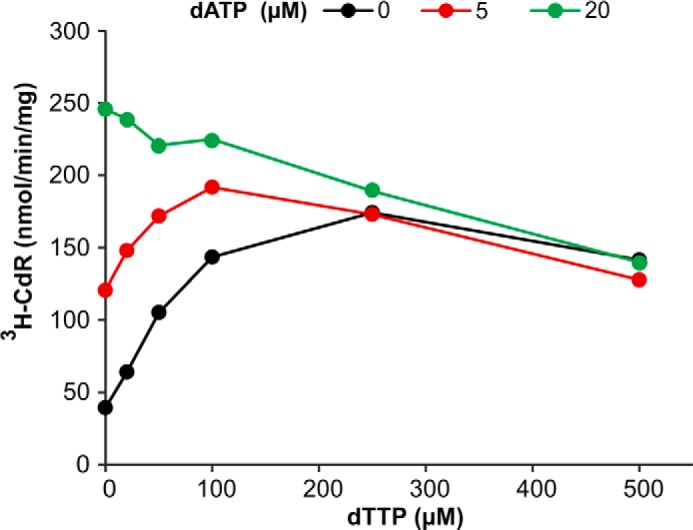
**Competition between dTTP and dATP for allosteric site 2 during dephosphorylation of dCTP by mouse SAMHD1.** We determined the influence of 5 or 20 μm dATP on the dephosphorylation of 30 μm [^3^H]dCTP by 50 ng of mouse SAMHD1 in the presence of increasing concentrations of dTTP as effector. All reactions were carried out in the presence of 20 μm GTP under the general conditions given in Fig. 2. The data demonstrate the superiority of dATP over dTTP as effector and the competition of the two dNTPs for allosteric site 2.

At allosteric site 2 we could demonstrate a competition between dATP and dTTP as effectors for the dephosphorylation of dCTP by mouse SAMHD1 (Fig. 7). In the absence of dATP the hydrolysis of dCTP depended on dTTP as effector with a *K_m_* constant of 50 μm (Table 2). On addition of increasing amounts of dATP, this nucleotide substituted for dTTP at allosteric site 2, and at 20 μm dATP became the only effector responsible for the activation. These results show that both dATP and dTTP bind to the same effector site and that dATP, in agreement with its smaller *K_m_* constant (Table 2), effectively competes with dTTP.

## DISCUSSION

The allosteric regulation of mouse and human SAMHD1 was incompletely understood when we started this investigation. Since then two publications about the crystal structure of the tetrameric protein in complex with effectors and substrate provided a precious key for the interpretation of the present results (13, 14). Each subunit of the protein contains, in addition to a substrate site, two allosteric sites (site 1 and site 2) with different properties, in excellent agreement with our present results. We show that the optimally activated mouse and human enzymes both have weak affinities for all four dNTP substrates, with similar large *K_m_* constants for each substrate (Table 1), but the mouse enzyme had slightly larger *k*_cat_ values. Both enzymes dephosphorylated single dNTPs also in the absence of effectors and were activated up to 4-fold by GTP in combination with various dNTPs. Thus, dNTPs are both substrates and effectors of SAMHD1. For each given dNTP the *K_m_* constant as effector was considerably smaller than its *K_m_* as a substrate (Tables 1 and 2), with the *K_m_* values of the mouse enzyme for dCTP possibly being an exception. A second general observation was that the mouse enzyme had much larger effector *K_m_* values than the human enzyme. From competition experiments we conclude that allosteric site 1 has a very high affinity for guanine nucleotides and that site 2 is specific for deoxyribose and binds dNTPs with lower but still high affinity.

We propose the following general scenario for the allosteric regulation of both enzymes. Site 1 accommodates GTP, the most abundant guanine nucleotide in the cell, stabilizing the tetrameric structure of the protein and resulting in the formation of site 2 (14, 22). Now dNTPs can bind to this site and activate SAMHD1. The activation occurs by binding of any dNTP to site 2 and affects the dephosphorylation of any dNTP. In some of our experiments this binding results in a self-activation of the reaction by the substrate. In live cells all four dNTPs are present as potential substrates and effectors. Thus, SAMHD1 activity will depend both on the intracellular concentration of the dNTP being a potential effector and on its *K_m_* constant at site 2. Small constants favor the dephosphorylation of low concentrations of intracellular dNTPs. The smaller *K_m_* constants of the human compared with the mouse enzyme provide a superior mechanism to maintain low intracellular dNTP concentrations.

How do the effector *K_m_* constants influence the *in vivo* function of SAMHD1? The *K_m_* of GTP for site 1 is low, and at the physiological concentrations of the nucleotide site 1 will be saturated by the effector and SAMHD1 will normally be a tetramer, ready to be activated by dATP, dGTP, or dTTP at site 2. SAMHD1 is expressed mainly in quiescent cells which contain very small dGTP pools, thus a major regulatory function of this nucleotide is unlikely. The intracellular concentrations of dATP and, in the case of the human enzyme, also dTTP lie well in the range covered by the *K_m_* constants of the two enzymes. We therefore believe that these two deoxynucleotides, and in particular dATP, are the most important regulators of SAMHD1 activity.

The concentrations of dNTPs are highly dependent on the proliferation state of cells (24, 25). They are high, up to 100 μm, to support nuclear DNA replication, but rapidly decline as replication is completed, and the R2 small subunit of ribonucleotide reductase disappears (24). Outside S phase and in noncycling cells the dNTP pools are small, decreasing to <1 μm for dGTP (23), but adequate for mitochondrial DNA maintenance and DNA repair (26). SAMHD1 plays a major role in the interplay between synthesis and degradation of dNTPs (1, 6). Loss of SAMHD1, either by gene targeting (20, 21), silencing (17), or regulated proteolysis (6), leads to an expansion of dNTP pools demonstrating that SAMHD1 restricts pool sizes. From the *K_m_* values reported here we can predict that in quiescent or differentiated cells SAMHD1 is not working at its maximum potential but still contributes significantly to the regulation of dNTP pools. The enzyme works outside S phase as a guardian of pool sizes by sensing dNTP fluctuations, *e.g.* due to variations of extracellular deoxyribonucleoside levels or due to invasion by viruses provided with their own dNTP synthesizing enzymes such as Herpes viruses. It was shown that SAMHD1 restricts not only HIV-I proliferation but also that of other human viruses, including Herpes (27, 28).

Returning to the different phenotypes of SAMHD1 deficiency in humans and mice, our present data demonstrate quantitative but not qualitative differences in the regulation of the two enzymes. Alone they are probably not sufficient to fully explain the different phenotypes of SAMHD1 deficiency in the two species. Other as yet unknown factors are likely involved. A solution for this problem requires further work with intact cells, but it will have to incorporate the results of our present investigation.

## Footnotes

3 The abbreviation used is:

SAMDH1: sterile α-motif/histidine-aspartate domain-containing protein 1.
